# Probable Coxsackievirus Infection in an Immunocompetent Adult With Genital and Oral Ulcerations Mimicking Herpes Simplex Virus Infection and Behçet’s Disease

**DOI:** 10.7759/cureus.113770

**Published:** 2026-08-01

**Authors:** Jean C Gutierrez-Silva, Nicole Fontanet-Gomez, Eugenio Grand Barrios, Parul Aneja, Anil Chopra

**Affiliations:** 1 Internal Medicine, St. Joseph's Hospital, BayCare Health System, Tampa, USA; 2 Infectious Diseases, St. Joseph's Hospital, BayCare Health System, Tampa, USA; 3 Infectious Diseases, Infectious Disease Consultants of West Florida, Tampa, USA

**Keywords:** coxsackievirus, disseminated viral infection, enterovirus, genital ulceration, mucocutaneous ulcers

## Abstract

Disseminated viral infections in immunocompetent adults are uncommon and may mimic autoimmune or bacterial conditions, including Behçet’s disease and disseminated herpes simplex virus (HSV) infection. We report a case of a 33-year-old immunocompetent woman who developed severe, painful vulvar ulcerations, oral ulcers, and a painful maculopapular rash involving both lower extremities that evolved into pustules. She was afebrile and hemodynamically stable but demonstrated a marked systemic inflammatory response, including leukocytosis (white blood cell count: 21.3×10⁹/L; 85% neutrophils) and elevated inflammatory markers (erythrocyte sedimentation rate: 42 mm/h; C-reactive protein: 15.4 mg/dL). Extensive infectious and autoimmune evaluation was unrevealing, and imaging showed no drainable pelvic fluid collection. She received empiric broad-spectrum antibiotics and was transitioned to valacyclovir for suspected disseminated HSV infection; leukocytosis improved rapidly with persistently sterile cultures. Subsequent serologic testing demonstrated elevated Coxsackievirus A and B titers (≥1:100), supporting an enteroviral etiology in the appropriate clinical context; however, the limitations of serologic testing preclude definitive confirmation of acute infection in the absence of paired titers or lesion-based polymerase chain reaction testing. She was transferred to a tertiary center for dermatologic evaluation due to the absence of local specialty services, where skin biopsy demonstrated nonspecific inflammatory changes with negative direct immunofluorescence, arguing against immune complex-mediated vasculitis. This case highlights a rare adult presentation of Coxsackievirus infection with prominent genital and oral ulcerations mimicking HSV and Behçet’s disease, underscoring the importance of considering viral etiologies in sterile inflammatory mucocutaneous eruptions to avoid unnecessary antimicrobial exposure.

## Introduction

Enteroviruses, including Coxsackieviruses, classically cause self-limited febrile illness and hand, foot, and mouth disease (HFMD) in children. However, adult infections are increasingly recognized and may present with more severe and atypical mucocutaneous manifestations than traditionally described, including disseminated involvement characterized by widespread cutaneous and mucosal disease extending beyond the classic palmoplantar distribution [1-3]. Outbreaks associated with Coxsackievirus A6 have demonstrated an expanded clinical spectrum, including vesiculobullous eruptions and widespread cutaneous involvement [1,2,4]. Early recognition of these atypical presentations is important to support antimicrobial stewardship by avoiding unnecessary broad-spectrum antibiotic and antiviral exposure.

Acute painful oral and genital ulcerations accompanied by systemic inflammation in an immunocompetent host can closely resemble disseminated herpes simplex virus (HSV) infection, sexually transmitted infections, drug eruptions, or inflammatory disorders, such as Behçet's disease, a chronic systemic vasculitis characterized by recurrent oral and genital ulcerations with potential ocular, vascular, and neurologic involvement [5,6]. This diagnostic overlap may prompt extensive testing and empiric antimicrobial therapy before viral etiologies are considered. We describe an immunocompetent adult presenting with painful oral and vulvar ulcerations and a pustular lower-extremity eruption initially concerning for disseminated HSV infection and Behçet's disease, later supported by elevated Coxsackievirus serologies.

Although several reports have documented atypical adult Coxsackievirus presentations, including widespread vesiculobullous eruptions, purpuric lesions, and eczema coxsackium, reports of adult enteroviral infection presenting with severe genital and oral ulcerations, pustular lower-extremity lesions, and a clinical picture closely resembling both disseminated herpes simplex virus infection and Behçet's disease have not been previously described [3].

## Case presentation

A 33-year-old immunocompetent woman with no significant past medical history presented with severe vulvar pain and mucocutaneous lesions. Symptoms began approximately one week prior with vulvar burning that progressed to severe, painful labial ulcerations. Over the subsequent 48 h, she developed painful oral mucosal ulcerations and a maculopapular rash involving both lower extremities that evolved into tender pustules. The eruption spared the trunk, palms, and soles. She reported malaise and chills prior to admission but was uncertain about documented fever.

She denied recent sexual activity, prior sexually transmitted infections, autoimmune disease, or similar dermatologic episodes. Recent travel included a trip to Toronto, though symptoms preceded travel. She was not immunocompromised and was not receiving immunosuppressive therapy.

On presentation, she was afebrile and hemodynamically stable. Physical examination revealed irregular vulvar and perineal ulcerations with whitish discoloration and no purulent drainage, along with multiple shallow, round-to-oval oral ulcers with erythematous borders involving the buccal mucosa, ranging from approximately 3 to 5 mm in diameter. The lower-extremity lesions consisted of a symmetric maculopapular eruption with pustular evolution (Figures 1, 2). No lymphadenopathy was appreciated.

**Figure 1:**
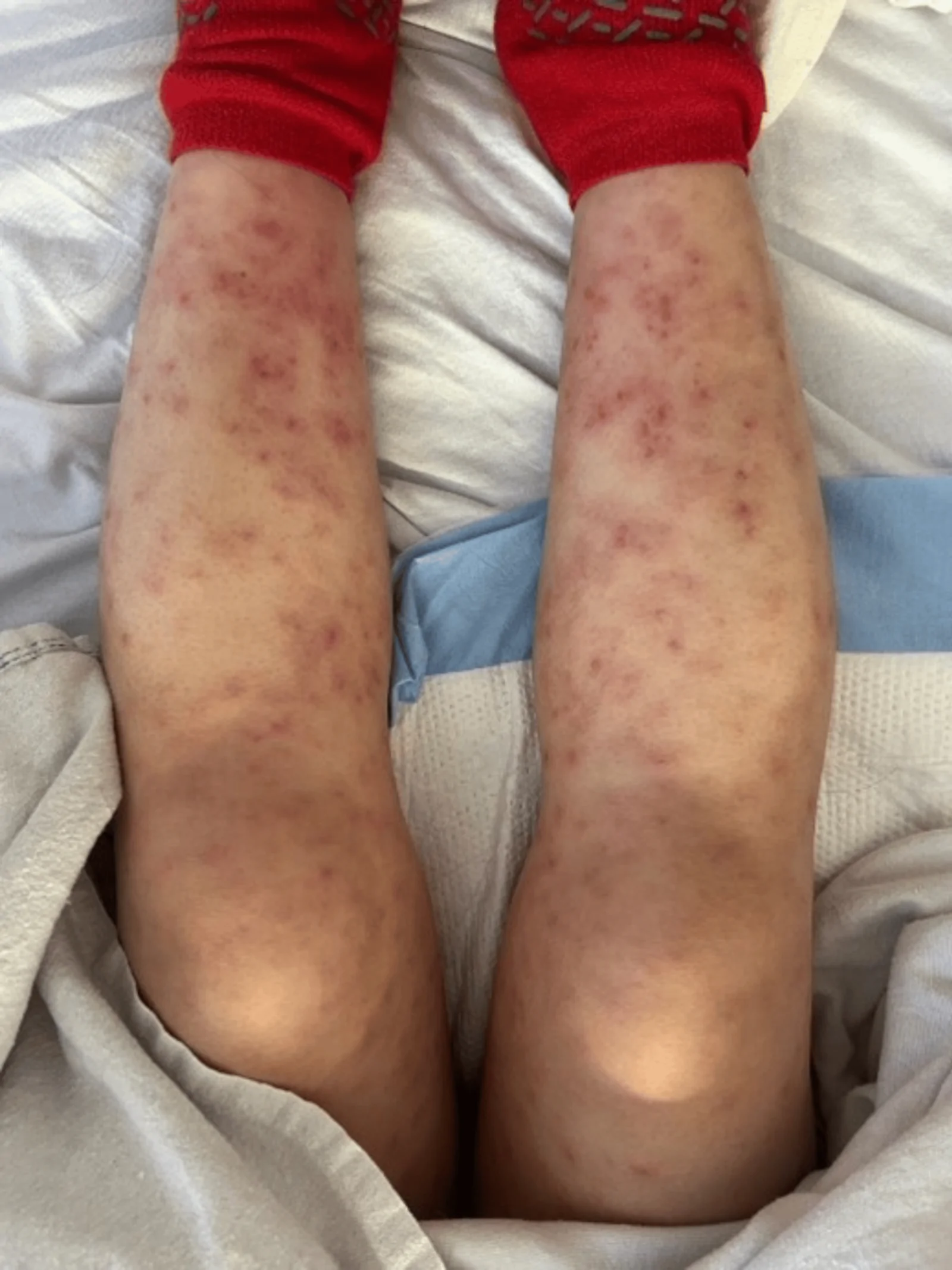
Cutaneous manifestations of suspected Coxsackievirus infection. Bilateral, symmetric papulopustular eruption involving the anterior shins and lower legs.

**Figure 2:**
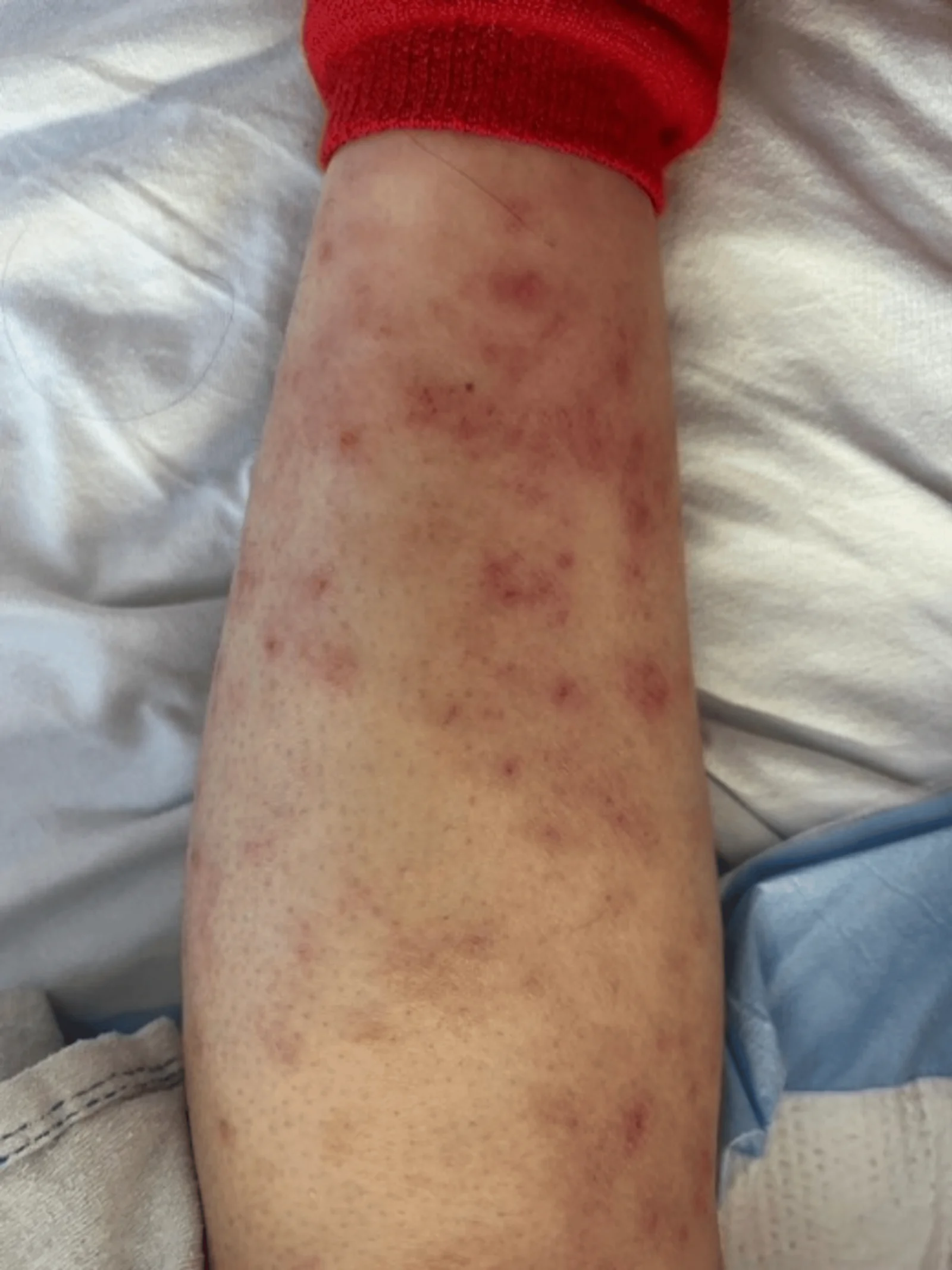
Close-up view of lesions on the anterior shin. Close-up view of lesions on the anterior shin demonstrating erythematous to violaceous papules and pustules on an erythematous base without vesiculation, ulceration, or necrosis.

Laboratory evaluation demonstrated marked leukocytosis (white blood cell count: 21.3×10⁹/L with 85% neutrophils) and elevated inflammatory markers, including an erythrocyte sedimentation rate of 42 mm/h and C-reactive protein of 15.4 mg/dL (Table 1). Renal and hepatic function were normal. Urinalysis showed pyuria; however, urine and blood cultures remained sterile. Computed tomography of the pelvis demonstrated an intrauterine device in situ without evidence of a drainable fluid collection.

**Table 1:** Serum and hematologic results.

Test	Result	Reference range/normal	Notes
WBC	21.3×10⁹/L (85% neutrophils)	4.0-10.5×10⁹/L	Elevated
ESR	42 mm/h	0-20 mm/h	Elevated
CRP	15.4 mg/dL	0.0-0.50 mg/dL	Elevated
Procalcitonin	0.10 ng/mL	<0.10 ng/mL	Slightly elevated

Given concern for severe bacterial infection in the setting of systemic inflammation, empiric therapy with vancomycin, meropenem, and clindamycin was initiated. Due to persistent concern for disseminated HSV infection, valacyclovir was added. Comprehensive infectious testing was negative, including HIV, treponemal serologies, gonorrhea and chlamydia testing, trichomonas testing, Candida polymerase chain reaction, and negative serum HSV-1/2 polymerase chain reaction. Autoimmune evaluation, including antinuclear antibody and HLA-B51 testing, was unrevealing (Table 2).

**Table 2:** Infectious and autoimmune testing results. HSV: herpes simplex virus; H: laboratory value above the normal reference range (high)

Test	Result	Reference/normal	Notes
Coxsackie A7 Ab	1:100 (H)	<1:100 (negative)	High
Coxsackie A9 Ab	1:100 (H)	<1:100 (negative)	High
Coxsackie B1 Ab	1:100 (H)	<1:100 (negative)	High
Coxsackie B2 Ab	Negative	<1:100 (negative)	Negative
Coxsackie B4 Ab	1:100 (H)	<1:100 (negative)	High
Coxsackie B5 Ab	1:100 (H)	<1:100 (negative)	High
Coxsackie B6 Ab	1:100 (H)	<1:100 (negative)	High
Coxsackie A16 Ab	1:100 (H)	<1:100 (negative)	High
Coxsackie A24 Ab	1:100 (H)	<1:100 (negative)	High
Chlamydia PCR	Negative	Negative	Negative
*Neisseria gonorrhoeae* PCR	Negative	Negative	Negative
Trichomonas PCR	Negative	Negative	Negative
Bacterial vaginosis PCR	Negative	Negative	Negative
Candida group PCR	Negative	Negative	Negative
*Candida glabrata/krusei* PCR	Negative	Negative	Negative
HSV‑1	Negative	Negative	Negative
HSV‑2	Negative	Negative	Negative
ANA	Negative	Negative	Negative
HLA‑B51	Negative	Negative	Negative

Over the subsequent three days, leukocytosis improved from 21.3 to 8.7×10⁹/L with continued clinical stability and persistently negative cultures (Table 3). Empiric antibacterial therapy was administered for approximately three days and discontinued following improvement in leukocytosis and persistently sterile cultures. Supportive care included topical analgesics and local wound care for the mucocutaneous lesions. Because dermatology services were unavailable locally, the patient was transferred to a tertiary care center for further evaluation and consideration of a biopsy. Subsequent serologic testing demonstrated elevated Coxsackievirus A (A7-A24) and B (B1-B6) titers (≥1:100), suggesting recent enteroviral exposure in the appropriate clinical context.

**Table 3:** Timeline of clinical course. IUD: intrauterine device; HSV: herpes simplex virus; GC/CT: *Neisseria gonorrhoeae*/*Chlamydia trachomatis*; BV: bacterial vaginosis; DIF: direct immunofluorescence

Timeline	Clinical event	Diagnostic workup	Therapeutic intervention
One week pre-admission	Vulvar burning begins; progressive labial ulcerations develop	-	-
48 h pre-admission	Oral mucosal ulcerations and bilateral lower-extremity maculopapular rash with pustular evolution; malaise and chills	-	-
Day 0 (hospital admission)	Afebrile, hemodynamically stable; exam reveals vulvar/perineal ulcerations, oral ulcers, symmetric lower-extremity papulopustular eruption; no lymphadenopathy	WBC: 21.3×10⁹/L (85% neutrophils), ESR: 42, CRP: 15.4, procalcitonin: 0.10; CT pelvis: IUD in situ, no drainable collection; urine/blood cultures sent	Empiric vancomycin, meropenem, clindamycin initiated
Day 0-1	-	HSV-1/2 PCR, HIV, treponemal serologies, GC/CT PCR, trichomonas PCR, Candida PCR, BV PCR sent; ANA, HLA-B51 sent	Valacyclovir added for suspected disseminated HSV
Day 1-3	Clinical stability; leukocytosis improves (WBC: 21.3 → 8.7×10⁹/L)	All infectious and autoimmune testing returns negative; cultures remain sterile	Antibacterial therapy discontinued
Day 3-4 (transfer)	Transferred to tertiary center for dermatologic evaluation (no local dermatology services)	-	-
Tertiary center admission	Dermatologic evaluation performed; transient bilateral ankle arthralgia with mild swelling develops	Skin biopsy of lower-extremity lesion obtained; Coxsackievirus A and B serologies sent	Supportive care
Post-discharge (follow-up)	-	Biopsy: nonspecific inflammation, no leukocytoclastic vasculitis, no viral cytopathic effect; DIF negative for all immunoreactants; Coxsackievirus A (A7, A9, A16, A24) and B (B1, B4, B5, B6) titers ≥1:100	-

Following the transfer, dermatologic evaluation was performed. A skin biopsy obtained from a representative lower-extremity lesion demonstrated nonspecific inflammatory changes without evidence of leukocytoclastic vasculitis or viral cytopathic effect. Frozen section confirmed adequate tissue sampling for direct immunofluorescence, which was negative for all immunoreactants, including IgA, IgG, IgM, C3, albumin, and fibrinogen, arguing against immune complex-mediated vasculitis. During that hospitalization, the patient also developed transient bilateral ankle arthralgia with mild swelling, consistent with an acute inflammatory process. No erosive changes or features suggestive of chronic inflammatory arthritis were identified. The patient was subsequently contacted for follow-up and provided the final pathological results, which confirmed these findings. At follow-up, the vulvar and oral ulcerations had resolved, and the lower-extremity eruption had faded without scarring. No recurrence of symptoms was reported.

Skin biopsy results

H&E sections show a skin punch biopsy with basketweave orthokeratosis, intracorneal collection of neutrophils, and normal epidermal maturation. Focal interface changes are identified. Within the superficial dermis, there is a perivascular inflammatory infiltrate composed predominantly of lymphocytes, with occasional neutrophils. There is a perifollicular inflammatory infiltrate with occasional neutrophils. Focally, a superficial blood vessel with thrombus formation is identified. Periodic acid-Schiff (PAS), Grocott’s methenamine silver (GMS), and Fite-Faraco stains, with appropriate controls, are negative for microorganisms. Multiple deeper sections were examined.

Frozen section shows an adequate tissue biopsy specimen for direct immunofluorescence. Direct immunofluorescence is negative for all immunoreactants, including IgA, IgG, IgM, C3, albumin, and fibrinogen.

## Discussion

Disseminated Coxsackievirus infections in immunocompetent adults are rare and can be diagnostically challenging due to overlap with more common infectious and inflammatory conditions. Although hand, foot, and mouth disease (HFMD) is classically considered a pediatric illness, adult cases are increasingly recognized and may present with atypical or severe mucocutaneous involvement [3-7]. Reported adult manifestations include vesiculobullous eruptions, eczema coxsackium, and widespread cutaneous disease, reflecting an expanded clinical spectrum beyond classic pediatric presentations.

In this case, the presence of painful oral and genital ulcerations with systemic inflammation appropriately raised concern for disseminated HSV infection and inflammatory disorders such as Behçet's disease. Disseminated HSV remains an important diagnostic consideration given its potential severity and need for prompt antiviral therapy [8]. Behçet's disease is characterized by recurrent oral and genital ulcerations with possible multisystem involvement; however, the absence of prior episodes, lack of ocular or neurologic findings, and negative autoimmune evaluation made a primary inflammatory disorder less likely in this patient [9,10]. Additionally, dermatopathological evaluation at the tertiary center demonstrated nonspecific inflammatory changes and negative direct immunofluorescence, arguing against immune complex-mediated vasculitis and further reducing concern for an underlying autoimmune vasculitis.

The clinical distinction among these entities can be further clarified by their characteristic features. Disseminated HSV typically presents with grouped vesicles on an erythematous base that evolve into shallow ulcerations, often with positive viral PCR or culture and characteristic multinucleated giant cells on biopsy. Behçet's disease is defined by recurrent episodes of oral and genital ulcerations, often accompanied by ocular inflammation, pathergy, or neurologic involvement, with biopsy typically showing neutrophilic vasculitis. In contrast, the present case demonstrated a single episode of oral and genital ulcerations with a pustular lower-extremity eruption, negative HSV PCR, negative autoimmune markers, no prior episodes, and biopsy findings showing nonspecific perivascular inflammation without vasculitis or viral cytopathic effect, a pattern more consistent with a self-limited viral process than with either HSV or Behçet's disease.

Beyond HSV and Behçet's disease, several other entities warrant consideration in the differential diagnosis of acute oral and genital ulcerations. Lipschütz ulcer is characterized by the sudden onset of painful, necrotic vulvar ulcerations in young women, often preceded by a flu-like prodrome, and has been linked to Epstein-Barr virus, cytomegalovirus, and Mycoplasma species. Like the present case, Lipschütz ulcers are a diagnosis of exclusion, demonstrate nonspecific histopathology, and resolve spontaneously within two to four weeks. However, Lipschütz ulcers typically present with deep necrotic ulcerations rather than the pustular lower-extremity eruption observed in our patient and are not typically associated with enteroviral infection. Mycoplasma pneumoniae-induced rash and mucositis (MIRM) is another important consideration, characterized by prominent oral, ocular, and urogenital mucositis with sparse cutaneous involvement, typically following respiratory symptoms. The absence of respiratory symptoms, negative findings for Mycoplasma, and the prominent pustular cutaneous eruption in our patient argued against MIRM. Erythema multiforme, which may present with oral and genital mucosal erosions and targetoid cutaneous lesions, was also considered; however, the absence of classic target lesions and the pustular rather than vesiculobullous morphology of the skin eruption made this diagnosis less likely. The clinical overlap among these entities underscores the diagnostic challenge posed by acute mucocutaneous ulcerations and the importance of a systematic approach to exclude infectious, inflammatory, and reactive etiologies [3,7].

Although Coxsackievirus serologic testing has limitations, including cross-reactivity and the inability to definitively confirm acute infection without paired titers or lesion-based polymerase chain reaction, elevated titers in the setting of a compatible clinical syndrome and exclusion of alternative etiologies support an enteroviral cause. Paired acute and convalescent titers were not obtained in this case, which would have strengthened the serologic evidence by demonstrating a fourfold rise in antibody levels. Additionally, lesion-based PCR was not performed for either enterovirus or HSV, as dermatology services and specialized virologic testing were unavailable at the initial facility; the patient required transfer to a tertiary center for biopsy alone. Notably, HSV-1 and HSV-2 PCR testing was performed on serum rather than from lesion swabs, which may reduce diagnostic sensitivity for mucocutaneous HSV infection, as lesion-based PCR is the preferred method for detecting active cutaneous or genital herpes simplex virus infection. The absence of definitive virologic confirmation, either through lesion PCR or paired serologies, represents the principal diagnostic limitation of this report. When available, lesion polymerase chain reaction testing may further improve diagnostic certainty.

This case underscores the importance of considering viral etiologies in immunocompetent adults presenting with sterile inflammatory mucocutaneous eruptions and systemic inflammatory responses. Early recognition of atypical enteroviral infections may facilitate timely antimicrobial de-escalation, reduce unnecessary broad-spectrum antibiotic exposure, and mitigate medication-related adverse effects, supporting antimicrobial stewardship efforts [11].

## Conclusions

Coxsackievirus infection, classically associated with pediatric hand-foot-and-mouth disease, can present atypically in immunocompetent adults with severe mucocutaneous involvement and a marked systemic inflammatory response. In this case, prominent genital and oral ulcerations with leukocytosis and elevated inflammatory markers closely mimicked disseminated HSV infection and inflammatory disorders such as Behçet's disease, creating substantial diagnostic uncertainty. The absence of positive bacterial cultures, negative herpes simplex virus testing, unremarkable autoimmune evaluation, and nondiagnostic biopsy findings, combined with elevated Coxsackievirus serologic titers in a compatible clinical context, supported but did not definitively confirm an enteroviral etiology. Recognition of this atypical presentation is important to avoid unnecessary antimicrobial exposure, reduce invasive diagnostic interventions, and support appropriate supportive care. Increased awareness of adult enteroviral infections may improve diagnostic accuracy and reinforce antimicrobial stewardship efforts in similar clinical scenarios.
